# Mortality after primary intracerebral hemorrhage in relation to post-stroke seizures

**DOI:** 10.1007/s00415-017-8573-1

**Published:** 2017-07-25

**Authors:** Danny Claessens, Kim Bekelaar, Floris H. B. M. Schreuder, Bianca T. A. de Greef, Mariëlle C. G. Vlooswijk, Julie Staals, Robert J. van Oostenbrugge, Rob P. W. Rouhl

**Affiliations:** 1 Department of Neurology, Maastricht University Medical Center+ (MUMC+), PO Box 5800, 6202AZ Maastricht, The Netherlands; 2 Cardiovascular Research Institute Maastricht (CARIM), Maastricht University, Maastricht, The Netherlands; 3 Academic Center for Epileptology MUMC+ and Kempenhaeghe, Heeze, Maastricht, The Netherlands; 4 School of Mental Health and Neurosciences (MHeNS), Maastricht University, Maastricht, The Netherlands; 5 Department of Neurology, Radboud University Medical Center, Nijmegen, The Netherlands

**Keywords:** Intracerebral hemorrhage, Seizures, Prognosis, Cohort studies

## Abstract

Seizures after intracerebral hemorrhage are repeatedly seen. Whether the development of seizures after intracerebral hemorrhage affects survival in the long term is unknown. This study aims to determine the relation between seizures (i.e., with and without anti-epileptic therapy) and long-term mortality risk in a large patient population with intracerebral hemorrhage. We retrospectively included patients with a non-traumatic ICH in all three hospitals in the South Limburg region in the Netherlands between January 1st 2004 and December 31st 2009, and we assessed all-cause mortality until March 14th 2016. Patient who did not survive the first seven days after intracerebral hemorrhage were excluded from analyses. We used Cox multivariate analyses to determine independent predictors of mortality. Of 1214 patients, 783 hemorrhagic stroke patients fulfilled the inclusion criteria, amongst whom 37 (4.7%) patients developed early seizures (within 7 days after hemorrhage) and 77 (9.8%) developed late seizures (more than 7 days after hemorrhage). Seizure development was not significantly related to mortality risk after correction for conventional vascular risk factors and hemorrhage severity. However, we found a small but independent relation between the use of anti-epileptic drugs and a lower long-term mortality (HR = 0.32, 95% CI 0.11–0.91). In our large population, seizures and epilepsy did not relate independently to an increased mortality risk after hemorrhage.

## Introduction

Intracerebral hemorrhage (ICH) accounts for roughly 10–20% of all strokes, and has a 10-year survival of only 24% [1–4]. Furthermore, survivors of an ICH have a 10% risk to develop seizures [5, 6]. These seizures may develop within seven days (i.e., early, acute symptomatic seizures), or later (i.e., late, remote symptomatic seizures) [7]. The development of seizures may inflict additional cerebral damage, and hence increase mortality risks after ICH. However, seizure development might also coincide with important risk factors for mortality (e.g., old age, stroke severity, cortical involvement, and alcohol consumption) [6, 8, 9].

Previous studies showed conflicting findings with regard to the relation between early and late seizures, and mortality, mostly based on differences in follow-up, definition of seizures, number of included patients, and inhomogeneous patient populations [6, 10, 12–18]. Furthermore, early, late, and recurrent seizures differ in pathogenesis and might also have a different relation with mortality [10, 11]. Because of the inconclusive evidence, we aimed at defining the long-term mortality risk after ICH in a large population, and relate this to post-stroke seizures. We hypothesized that the development of seizures negatively influences the survival of patients after ICH.

## Methods

The present study is conducted in accordance with the Strengthening the Reporting of Observational Studies in Epidemiology (STROBE statement) [19].

### Participants and study design

We conducted a retrospective cohort study in all three hospitals in the South Limburg region of the Netherlands (i.e., hospitals of Maastricht, Sittard, and Heerlen). In agreement with the Dutch legislation, approval of the medical ethical committees was not necessary. We included all patients with a non-traumatic ICH, older than 18 years, between January 1st 2004 and December 31st 2009. All intracerebral hemorrhages were confirmed with at least CT-imaging [20]. We excluded secondary ICH and hemorrhagic transformation of intracerebral infarcts, non-parenchymatous hemorrhage (e.g., subdural, epidural, subarachnoid, and primary intraventricular hemorrhage), or hemorrhage associated with a brain tumor or encephalitis. We also excluded patients who did not survive the first seven days to prevent information bias, as these patients are not at risk of suffering from late seizures. Furthermore, we excluded patients with pre-existing epilepsy and concurrent use of anti-epileptic drugs, as assessed from records from previous out-patient department visits, to focus on hemorrhage-associated seizures.

### Variables, data sources and measurements

From hospital electronic patient records, especially looking for entries by neurologists, we retraced the occurrence of possible seizures both during hospitalization and during post-hospitalization out-patient visits and emergency department contacts in the same three hospitals by extracting reports from the treating neurologists. These possible seizures were assessed by two independent neurologists specialized in epilepsy (MV, RR). Criteria used for the assessment were those for seizures with generalized onset (e.g., bilateral uncontrolled movements, tonic posture, and/or loss of consciousness) and seizures with focal onset (e.g., hallucinations or cognitive deficits, and motor or sensory symptoms with evidence of seizure progression), and classified with the 2014 and 2017 definitions of the International League Against Epilepsy (i.e., ILAE) [21, 22]. Epilepsy was also defined according to ILAE guidelines [21, 22]. In case of doubt, events were not classified as epileptic seizures. We did not routinely use an electroencephalogram (EEG), we diagnosed seizures purely clinically. As a cut-off point for early seizures (ES) we used seven days, and after seven days we classified seizures as late seizures (LS) [23]. In case patient had both early and late seizures they would be defined as recurrent seizures, but these were analyzed as late seizures to maintain sample size.

We obtained the following variables: age at onset of the hemorrhage, gender, prior cerebrovascular disease (i.e., either ICH, ischemic stroke, or transient ischemic attack), vascular comorbidities (i.e., coronary heart disease, hypertension, diabetes mellitus, hypercholesterolemia, peripheral vascular disease, as determined by previous diagnosis in hospital records), National Institute of Health Stroke Scale at admission (NIHSS below 8, NIHSS 8-14, NIHSS above 14; grouped as described by Adams et al. [24]), location of hemorrhage (i.e., lobar, deep or infratentorial), septum shift, use of anticoagulants/antiplatelet drugs (i.e., either one, or combinational therapy), and recurrence of hemorrhage during follow-up (i.e., a second hemorrhage was not recorded as new hemorrhage). Furthermore, prior statin use, use of anti-epileptic drugs (AEDs), number of seizures before and after start of AEDs, and response to AED therapy were obtained. Municipal inhabitant registry was used to record all-cause mortality status for all patients, which is a very reliable method in the Netherlands to assess mortality as all deceased are registered by medical professionals, with the latest search dating March 14th 2016.

### Statistical analyses

Pearson Chi-square tests were performed to assess differences amongst seizure groups (no seizures, ES, or LS) in categorical data, as was done for continuous data with the use of the Student’s *t* test for normally distributed data and the Mann–Whitney *U* test for non-normally distributed data.

Mortality in the different seizure groups was analyzed with Kaplan–Meier analyses and log-rank tests, whereas we used Cox proportional hazard’s multivariable analysis to assess independent predictors of mortality. All variables were univariately analyzed and included in the multivariate analyses when significant or previously proven relevant. Cox multivariate analyses were not only performed for the complete follow-up, but also for fixed intermediate intervals (i.e., 2.5, 5, 7.5, and 10 years after onset of hemorrhage). This was done to analyze the association between seizure development and mortality over time (i.e., whether seizures or other predictors are variably associated with altered survival rates for different years after ICH). We censored patients that did not reach the primary endpoint (mortality) before the last follow-up, dating March 14th 2016 (end-of-study censoring). Association measures were expressed as hazard ratios (HR), with 95% confidence intervals. *p* values are provided for indication of significance, set at a value of *p* ≤ 0.05. All statistical methods were performed using SPSS (SPSS inc. Version 23.0, Chicago, Illinois).

## Results

### Participants

**Fig. 1:**
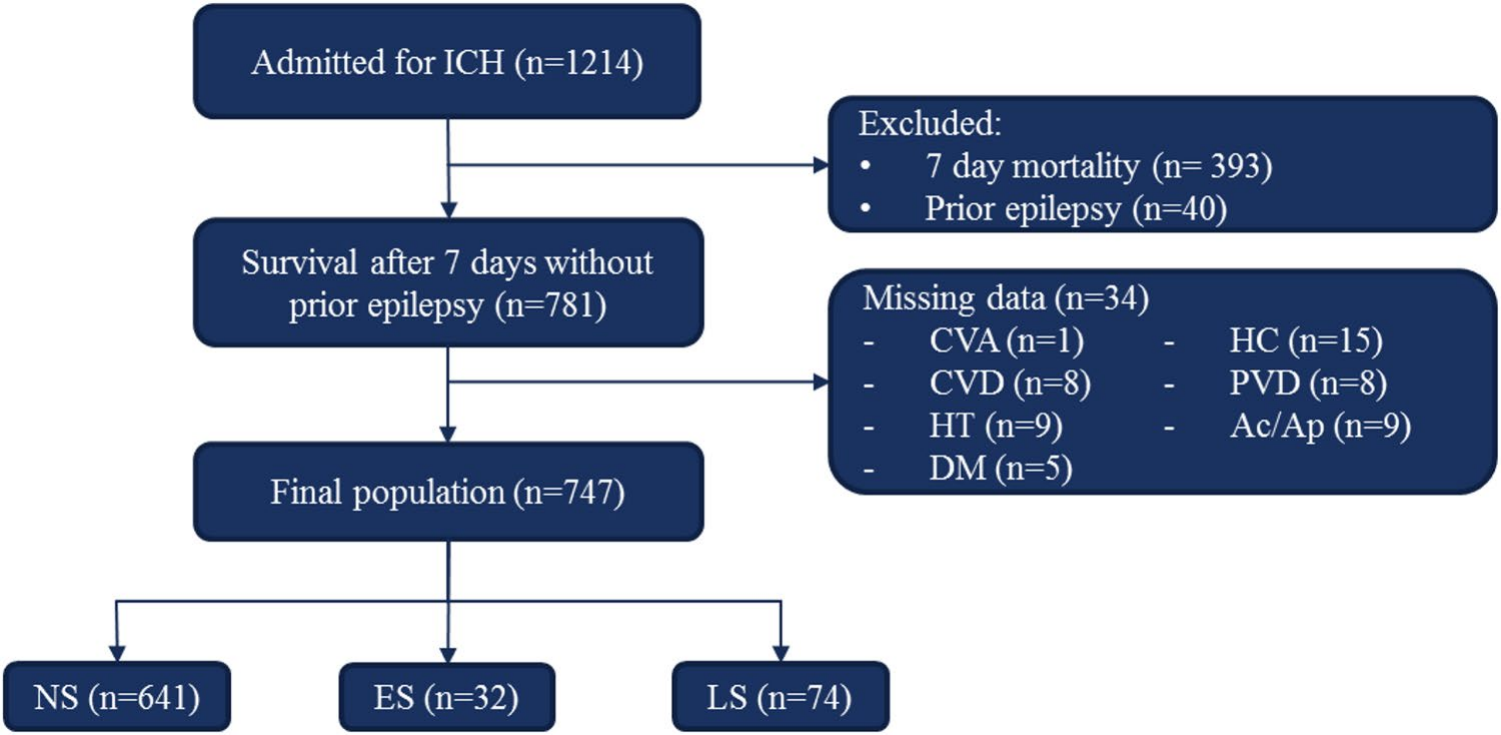
Selection process as applied in the study. Exclusion was based on mortality within the first 7 days, and second, on missing data of medical history, given per variable. The final population was divided in three groups, group sizes given. *ICH* intracerebral hemorrhage, *stroke* both ischemic as hemorrhagic, *CVD* cardiovascular disease, *HT* hypertension, *DM* diabetes mellitus type 2, *PVD* peripheral vascular disease, *Ac/Ap* anticoagulants/antiplatelet drugs, *n* number of patients either excluded or still present in the study, *NS* no seizures, *ES* early seizures, *LS* late seizures

We identified 1214 ICH patients in this retrospective study, and entered these into the selection process (Fig. 1). We excluded 393 patients who died within seven days after onset of the hemorrhage, 40 because of prior history of epilepsy, and 34 because of missing data: cerebrovascular disease (*n* = 1), cardiovascular disease (*n* = 8), hypertension (*n* = 9), diabetes (*n* = 5), hypercholesterolemia (*n* = 15), peripheral vascular disease (*n* = 11), and use of anticoagulants (*n* = 9) (Fig. 1). The remainder of 747 patients were used for all analyses. 641 (86%) patients did not develop seizures, 32 (4%) developed only early seizures, whereas 74 (10%) developed late seizures. Of these 74 patients, 29 had developed early seizures previously. These patients with recurrent seizures were analyzed as late seizures to maintain sample size. EEG-confirmation was available in 26 (20%) patients with late seizures, but none of the early seizures were EEG confirmed. Patients that developed late seizures mostly received AED therapy (93%). The follow-up for seizures and survival status had a median duration of 4.8 years (interquartile range of 0–12.6 years). Baseline characteristics of the study population are shown in Table 1.

**Table 1:** Baseline study characteristics

Variable	NS (*n* = 641)	ES (*n* = 32)	LS (*n* = 74)	*p* value
Age at hemorrhage in years	72.7 (71.7–73.7)	67.9 (62.6–73.1)	66.2 (62.9–69.5)	<0.001*
Gender (male)	339 (52.9%)	17 (53.1%)	43 (58.1%)	0.695
Cerebrovascular disease	175 (27.3%)	7 (21.9%)	18 (24.3%)	0.701
Cardiovascular disease	142 (22.2%)	6 (18.8%)	10 (13.5%)	0.214
Hypertension	392 (61.2%)	17 (53.1%)	38 (51.4%)	0.194
Diabetes mellitus	102 (15.9%)	4 (12.5%)	9 (12.2%)	0.628
Hypercholesterolemia	239 (37.3%)	14 (43.8%)	26 (35.1%)	0.699
Peripheral vascular disease	52 (8.1%)	1 (3.1%)	8 (10.8%)	0.411
NIHSS at admission				0.780
NIHSS < 8	371 (57.9%)	21 (65.6%)	40 (54.1%)	
NIHSS 8–14	144 (22.5%)	6 (18.8%)	16 (21.6%)	
NIHSS > 14	126 (19.7%)	5 (15.6%)	18 (24.3%)	
Location of hemorrhage				<0.001*, **
Lobar	256 (40.0%)	29 (90.6%)	61 (82.4%)	
Deep	326 (50.8%)	3 (9.4%)	10 (13.5%)	
Infratentorial	59 (9.2%)	0	3 (4.1%)	
Volume of hemorrhage in cm^3^	7.7 ± 21.6	6.7 ± 20.8	21.7 ± 30.4	0.012***
Septum shift mm	0 ± 3.2	0 ± 3.4	0.7 ± 3.7	n.s.
Cause of hemorrhage	57 (8.9%)	5 (15.6%)	12 (16.2%)	0.074
Use of anticoagulants/antiplatelet drugs	339 (52.9%)	12 (37.5%)	35 (47.3%)	0.172
ICH recurrence	58 (9.0%)	2 (6.3%)	13 (17.6%)	0.052
Mortality status at end of follow-up				0.964
Deceased	400 (62.4%)	20 (62.5%)	45 (60.8%)	
Surviving	241 (37.6%)	12 (37.5%)	29 (39.2%)	
EEG alterations	N.A.	0	20 (26%)	N.A.
AED use	0	0	72 (93%)	N.A.
Seizure free before AED	N.A.	N.A.	36 (49%)	N.A.
Seizure free after AED	N.A.	N.A.	31 (42%)	N.A.

Study characteristics specified per group (i.e., No seizures (*NS*), early seizures (*ES*) or late seizures (*LS*), mean ± 95% confidence interval (*CI*) for normally distributed continuous variables (i.e., age), median ± interquartile range (IQR) (i.e., for surface and septum shift). Significantly different groups (i.e., *p* < 0.05) were found for the comparisons NS versus LS (*), NS versus ES (**), or LS versus ES (***)

*NIHSS* National Institutes of Health Stroke Scale, *n.s.* not significant in any comparison, *N.A.* not applicable

### Outcome data

**Fig. 2:**
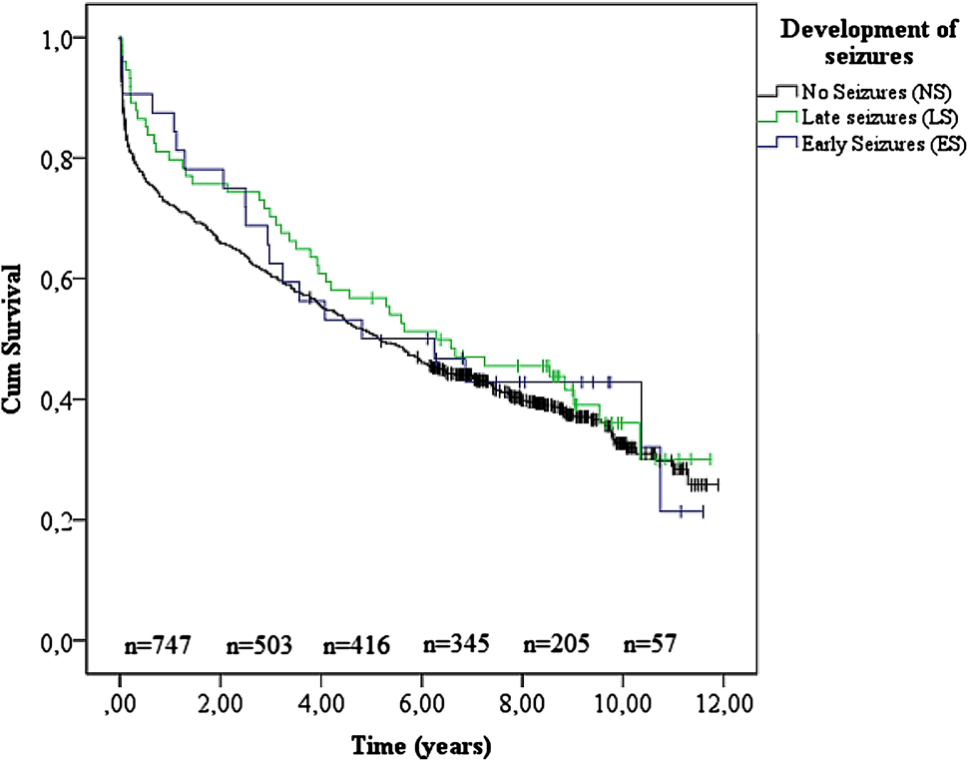
Kaplan–Meier analysis. Cumulative survival time analysis (unadjusted), specified on seizure development (i.e., NS, ES, or LS)

During follow-up, 465 (62%) patients died. 400 out of 641 (62%) died in the NS group, 20 out of 32 (54%) in the ES group, and 45 out of 74 (61%) in the LS group. See Fig. 2 for the Kaplan–Meier plots. The cumulative survival did not significantly differ between NS, ES, or LS (Log-rank *p* value 0.58).

### Predictors of post-hemorrhage mortality

We found that relevant predictors of mortality after ICH were higher age, male gender, peripheral vascular disease, higher NIHSS scores, and use of anticoagulants (i.e., no differences between anticoagulants or antiplatelet drugs were found) (Table 2). Factor is no longer significant in any time interval if statin use is included in the analyses. An additional significant predictor of mortality, only in the long term (i.e., 7.5 and 10 years), was diabetes mellitus (Table 2).

**Table 2:** Multivariable (Cox) analysis; predictors of late mortality

	2.5 years censored (*n* = 403)	5 years censored (*n* = 323)	7.5 years censored (*n* = 267)	10 years censored (*n* = 243)	Full follow-up* censored (*n* = 238)
	HR	HR	HR	HR	HR
Age at hemorrhage (years)	1.06 (1.05–1.08)	1.08 (1.06–1.09)	1.07 (1.06–1.09)	1.08 (1.06–1.08)	1.08 (1.06–1.09)
Gender (male)	1.44 (1.09–1.83)	1.46 (1.15–1.86)	1.35 (1.08–1.68)	1.35 (1.09–1.67)	1.33 (1.07–1.64)
History of cerebrovascular disease	n.s.	n.s.	n.s.	n.s.	n.s.
History of cardiovascular disease	n.s.	n.s.	n.s.	n.s.	n.s.
History of hypertension	n.s.	n.s.	n.s.	n.s.	n.s.
History of diabetes mellitus	n.s.	n.s.	1.40 (1.07–1.84)	1.37 (1.05–1.79)	1.38 (1.06–1.79)
History of hypercholesterolemia**	0.68 (0.50–0.91)	0.75 (0.58–0.96)	0.64 (0.50–0.81)	0.67 (0.53–0.84)	0.69 (0.55–0.87)
History of peripheral vascular disease	1.70 (1.07–2.71)	1.78 (1.20–2.65)	1.81 (1.24–2.63)	1.87 (1.30–2.70)	1.87 (1.29–2.69)
NIHSS at admission	1.78 (1.52–2.09)	1.55 (1.35–1.78)	1.55 (1.36–1.77)	1.58 (1.39–1.79)	1.58 (1.39–1.79)
Known cause of hemorrhage	n.s.	n.s.	n.s.	n.s.	n.s.
Use of anticoagulants	1.56 (1.15–2.11)	1.47 (1.13–1.90)	1.56 (1.22–1.98)	1.45 (1.15–1.82)	1.43 (1.14–1.80)
First recurrence	n.s.	n.s.	n.s.	n.s.	n.s.
Development of early seizures	n.s.	n.s.	n.s.	n.s.	n.s.
Development of late seizures	n.s.	n.s.	n.s.	n.s.	n.s.
AED use***	n.s.	n.s.	n.s.	n.s.	n.s.

Hazard ratios (±95% CI) for significant covariates in the multivariate Cox survival analysis for the comparison of survival between patients that do not develop seizures (no seizures *NS*), patients that develop seizures within 7 days after the time of hemorrhage onset (early seizures), and patients that develop seizures more than 7 days after the time of hemorrhage onset (late seizures *LS*)

*HR* hazard ratio, *n.s.* non-significant, *AED* anti-epileptic drug

* Maximal follow-up up to 12 years (*n* = 27; 3.6%)

** No longer significant in any interval if statin use is included

*** Only patients who developed late seizures were given AED

Interestingly, amongst patients with late seizures, the use of AEDs did relate to lower mortality rates in the 7.5- and 10-year intervals [HR 0.32 (0.11–0.91)]. In this group, there was no relation between mortality and the number of seizures before initiation of medication, and reaction to anti-epileptic drugs. However, the number of patients with LS at this long follow-up interval was relatively low (*n* = 35 and *n* = 7 for 7.5 and 10 years, respectively).

## Discussion

Our study underlines that seizures after ICH do not independently relate to higher late mortality risks after follow-up up to 10 years. Mortality was associated with higher age, male gender, peripheral vascular disease, higher NIHSS scores, and anticoagulant/antiplatelet drug use. Mortality in the long term (i.e., 10 years) was also determined by diabetes mellitus. Overall, we found that 10-year survival after ICH was 37% in our population, which was not significantly influenced by development of early or late seizures.

These results are in line with most [6, 10 13–15, 18], but not all [12, 16, 17] studies. These studies showed an increased or decreased risk of mortality in patients with seizures. Take note that these study populations were composed of both ischaemic stroke (IS) and ICH patients, and extrapolation should be done carefully though no differences between IS and ICH were mentioned. However, these previous studies did have significantly shorter periods of follow-up, and none of these studies found seizures to be an independent risk factor of mortality in multivariable analyses [12, 16, 18]. The one study that did indicate a significant independent relation, and showed a higher mortality rate in the seizure group, assessed a heterogeneous study population of supposedly vascular events (including ischemic stroke, transient ischemic attack and ICH) at a young age. However, authors did not specifically include ICH patients, and they did not exclude patients not surviving the first seven days after stroke [17]. Up and till now, studies with a long-term follow-up, specifically for ICH patients, are lacking to the best of our knowledge. Therefore, our study in ICH patients with a large sample size, long-term follow-up, and a selection process designed to study the long-term relation between seizures and mortality adds significantly to the existing reports. Our finding that mortality is not influenced by seizure development, but is mostly determined by age, stroke severity, and vascular risk factors might imply that patients may benefit more from management of these factors, than from management of the seizures.

AED use did correlate, independently from seizure frequency, with lower mortality risks in patients with late seizures 7.5 years after hemorrhage. This correlation weakened in the 10-year interval and disappeared in the full follow-up, probably due to the limited number of patients that already reached that time point. This might suggest that AED therapy improves long-term survival after ICH, though not likely due to lowered seizures frequencies. There are, however, some caveats, such as the small number of patients with late seizures that did not receive AED therapy (*n* = 5). Also, discrepancies in patient treatment may play a role in this as patients with worse clinical conditions might not have been treated equally, or even have not been treated at all. Though, Gilad et al. found a better neurological outcome after treatment with valproate in patients surviving an ICH, which, in combination with our findings, suggests a (weakly) positive effect of AED on recovery and prognosis after ICH [25]. This finding suggests that survival rates might benefit from AED therapy, but further research is needed to provide sufficient evidence.

Our study has some limitations. First, despite our large population, only 14% of participants developed seizures, which somewhat limits the power of this study. Also, there is bias due to the retrospective design, though efforts have been made to reduce the impact: we minimized the amount of missing data using diagnosis codes and hospital stroke registries. Furthermore, defining seizures retrospectively are another possible source of bias. Patients may underreport focal seizures or may not seek medical attention or overreport, for example, syncopes as seizures. Furthermore, subclinical seizures are missed because EEG monitoring was not routinely performed, which might lead to underestimation of the frequency of seizure development. As a consequence of the retrospective design, only survival status could be monitored in the period of follow-up after hospital discharge. Details on seizure severity and post-hospitalization comorbidities, which could underlie the association between AED therapy and decreased mortality, are not known. Furthermore, patients with worse prognosis might not have been treated at all. As all of these data are not available, these results have to be interpreted with caution.

Notwithstanding these limitations, the strengths of our study remain the large sample size allowing enough patients to develop early or late seizures, combined with the follow-up allowing sufficient time to develop late seizures and determine the long-term survival rates. Also, exclusion of patients who deceased during the first seven days allows to draw conclusions concerning the mortality risk based on seizure development, without interference of mortality risks primarily due to the hemorrhage itself.

In conclusion, seizure development does not relate to the mortality risk after ICH in our population. However, mortality mostly relates to age, stroke severity and vascular comorbidities and anticoagulant/antiplatelet use, and therefore, these prognostic factors are of value when discussing treatment and prognosis after ICH.
